# Phage characterization analysis in respiratory samples from infected patients based on metagenomic next-generation sequencing

**DOI:** 10.3389/fcimb.2026.1779296

**Published:** 2026-03-12

**Authors:** Yuyan Huang, Qingqing Cai, Yingying Chen, Direerba Amutijiang, Yihan Lu, Weifeng Huang, Ling Li

**Affiliations:** 1 School of Public Health, Southern Medical University, Guangzhou, China; 2 School of Basic Medical Sciences, Southern Medical University, Guangzhou, China; 3 Genoxor Medical Science and Technology Inc., Shanghai, China; 4 Department of Epidemiology, Ministry of Education Key Laboratory of Public Health Safety, Fudan University School of Public Health, Shanghai, China; 5 Shanghai Institute of Infectious Disease and Biosecurity, Fudan University, Shanghai, China; 6 Department of Critical Care Medicine, Shanghai Xuhui Central Hospital, Zhongshan-Xuhui Hospital, Fudan University, Shanghai, China; 7 Guangdong Food and Drug Vocational College, Guangzhou, China

**Keywords:** bronchoalveolar lavage fluid, metagenomic next-generation sequencing (mNGS), phage, respiratory tract infection, sputum

## Abstract

**Background:**

Respiratory tract infections are common infectious diseases, with microbial dysbiosis closely linked to clinical outcomes in the host. As key regulators of bacteria, phages can influence the structure and stability of microbial communities by infecting host bacteria. Metagenomic next-generation sequencing (mNGS) enables comprehensive analysis of phage community characteristics in clinical samples.

**Methods:**

This study included 6,404 clinical samples, comprising 4,837 bronchoalveolar lavage fluids (BALF) and 1,567 sputum samples, for metagenomic next-generation sequencing (mNGS), while collecting patient demographics, sample types, mNGS results, and clinical outcomes. Host-derived sequences were removed post-sequencing and aligned against viral reference databases. Phage community structures across sample types were assessed using alpha and beta diversity metrics. Spearman correlation analysis explored associations between phages and bacteria. Further bioinformatics analysis was performed on 194 samples, including viral sequence assembly and identification using SPAdes, VirSorter2, and PhaMer; CD-HIT clustering and redundancy removal; CheckV quality assessment; PhaTYP lifestyle prediction; Prodigal protein gene annotation; and BLASTP alignment against the CARD database to screen for phage resistance genes.

**Results:**

The sputum and BALF groups exhibited comparable richness, diversity, and evenness, yet their community structures differed significantly. Intensive Care Unit (ICU) admission status was closely associated with reduced phage community diversity and significant alterations in community structure, and the abundance distribution of several phage families (*Peduoviridae*, *Autoscriptoviridae*, *Casjensviridae*, *Demerecviridae*) also changed significantly. Additionally, the phage community structure in sputum samples was significantly associated with patient clinical outcomes. Correlation analysis demonstrated that the *Aliceevansviridae* family in sputum samples had extensive positive associations with various bacteria. After assembly, 69.5% of pOTUs were predicted to be temperate phages, and 28.9% were predicted to be virulent phages; moreover, the vast majority (99.2%) of phage sequences showed low similarity to antibiotic resistance genes.

**Conclusion:**

This study identifies distinct phage community characteristics across respiratory sample types and reveals that ICU patients exhibit reduced phage diversity and markedly altered community structures. Furthermore, the phage composition in upper respiratory tract samples shows a clear relationship with patient prognosis, providing new insights into respiratory infection microecology.

## 1 Introduction

Respiratory tract infections are among the most common infectious diseases encountered in clinical practice, affecting both the upper and lower respiratory tracts and caused by various microorganisms including bacteria, viruses, and fungi (Yin et al., 2024; Reyes et al., 2025). Not only are they a major cause of sepsis—with the in-hospital mortality rate of severe community-acquired pneumonia (CAP) reaching as high as 20%–30% (Murray et al., 2022)—but CAP also has a particularly significant impact on vulnerable populations such as the elderly and immunocompromised. Currently, it is the infectious syndrome with the highest burden related to antimicrobial resistance (Sharma et al., 2017; Pfeifer and Rocha, 2024). While the primary pathogen varies, a resident bacterial microenvironment exists in the respiratory tract of these patients (Man et al., 2017; Klingensmith and Coopersmith, 2023). Research suggests that phages within this ecosystem actively interact with bacteria, influencing bacterial abundance, metabolism, and antibiotic resistance (Peng et al., 2024; Pfeifer and Rocha, 2024).

As a key factor regulating bacterial communities, the role of phages in the respiratory tract microbiome is gaining increasing attention (Khosravi et al., 2024). Phages are viruses that specifically infect and lyse bacteria and are regarded as the most abundant and diverse entities in the biosphere, with an estimated 10³¹ phages globally (Sharma et al., 2017). Structurally, phages consist of a protein capsid enclosing their nucleic acid; they recognize and bind to specific receptors on the surface of host bacteria, inject their genetic material into the bacterial cell, and utilize the bacteria’s metabolic machinery for replication and assembly, ultimately leading to bacterial lysis and the release of new phage particles (Watson et al., 2021; Strathdee et al., 2023). Previous studies have shown that phages are core members of the respiratory virome, where the class *Caudoviricetes* (encompassing the former families *Siphoviridae*, *Myoviridae*, and *Podoviridae*) and the family *Microviridae* are typically the most abundant taxa (Choi et al., 2021; Ferravante et al., 2022b; Conradie et al., 2024). These populations are not only highly abundant but also directly influence pulmonary microecological stability by shaping the structure of bacterial communities (Li R. et al., 2024). In infectious settings, increased abundance of certain phages has been found to positively correlate with disease severity (Bajaj et al., 2021; Ferravante et al., 2022a). However, to date, the specific roles of the virome—particularly phages—in driving respiratory infections remain elusive.

In recent years, metagenomic next-generation sequencing (mNGS) has developed rapidly (Zhao et al., 2024). Utilizing high-throughput sequencing to detect all microbial DNA and RNA in a specimen, mNGS has propelled advances in virological diagnostics and research, enabling unbiased detection and comprehensive analysis of viral communities in diverse clinical samples (Huang et al., 2023; Tan et al., 2024). Traditional diagnostic methods often lack the sensitivity and broad spectrum needed to detect a wide range of viral pathogens, especially in clinical samples such as bronchoalveolar lavage fluid (BALF) and sputum (Kitsios, 2018; He et al., 2025). In contrast, mNGS can not only analyze the compositional makeup of microorganisms but also retrieve massive amounts of phage information from biological samples (Chen et al., 2022; Li et al., 2022).

Among clinical samples from the respiratory tract, bronchoalveolar lavage fluid and sputum are commonly used sample types for viral detection. BALF is collected from the lower respiratory tract and can directly reflect the true microenvironmental status of lower respiratory tract infections. It is mainly used in the diagnosis of diseases such as tuberculosis, opportunistic infections, and lung cancer (Li J. et al., 2024; Liu et al., 2025). In contrast, sputum is collected from the upper respiratory tract and may contain mixed secretions from the oral cavity and pharynx. It is a routine specimen for pathogen detection in respiratory tract diseases (Huffnagle et al., 2017; Peng et al., 2020).

In healthy adults, the upper and lower respiratory tracts differ in anatomy and microbial load but share a broadly continuous core microbiota (Niculescu et al., 2025). High-throughput 16S rRNA gene and metagenomic surveys show that *Streptococcus*, *Prevotella*, *Veillonella* and *Neisseria* dominate the upper airways (Mancabelli et al., 2022; Bassis et al., 2015; Gioula et al., 2018), whereas the lower tract has very low biomass and a relatively stable community largely shaped by selective immigration of these commensals, with lower overall diversity and richness (Belizário et al., 2023; Niculescu et al., 2025). When bacterial respiratory infection occurs, this balance is disrupted and commensal taxa decline, while classical pathogens such as *Streptococcus pneumoniae*, *Haemophilus influenzae* and *Staphylococcus aureus* often predominate in both upper and lower airways (de Steenhuijsen Piters et al., 2016; Jumanov et al., 2021; Jones et al., 2025); *Klebsiella pneumoniae*, *Pseudomonas aeruginosa* and *Acinetobacter baumannii* are particularly associated with severe lower respiratory disease (Pettigrew et al., 2021; Jones et al., 2025). As bacteriophages require bacteria as hosts, these site-specific commensal and pathogenic communities are expected to shape the composition and dynamics of the respiratory phageome and are therefore essential for interpreting phage–bacteria interactions in respiratory infection (Alseth et al., 2019; Pfeifer and Rocha, 2024).

In this study, we retrospectively analyzed 6,404 clinical respiratory samples subjected to metagenomic next-generation sequencing. The study objectives included: (i) systematic analysis of phage diversity and taxonomic composition in both sample types; (ii) comparison of phage community diversity and distinct features between Intensive Care Unit (ICU) and non-ICU patients; (iii) evaluation of whether phage composition is related to patient prognosis; and (Sharma et al., 2017) characterization of phage lifestyles and the distribution of antibiotic resistance genes in clinical infection samples.

## 2 Methods

### 2.1 Study design

This study retrospectively analyzed 6,404 samples from patients with confirmed infections, comprising 4,837 bronchoalveolar lavage fluid (BALF) samples and 1,567 sputum samples. These samples were collected between January 2021 and December 2023 from 156 hospitals in 21 provinces and municipalities in China. In this clinical setting, mNGS was employed as an adjunctive tool for the comprehensive identification of pathogens in patients with suspected respiratory infections.

The inclusion criteria were as follows (Jones et al., 2025): (i) Age ≥18 years and ≤80 years, regardless of gender; (ii) Clinical manifestations consistent with respiratory tract infection (such as cough, sputum production, fever, dyspnea, etc.); (iii) Imaging findings (such as chest X-ray or CT) suggesting respiratory tract infection (such as pneumonia, bronchitis, etc.); (iv) Laboratory tests (such as routine blood tests, CRP, PCT, etc.) supporting the diagnosis of infection; (v) Confirmation of infectious pathogens by mNGS or traditional methods; (vi) Signed informed consent.

The exclusion criteria were as follows (Jones et al., 2025): (i) Incomplete clinical data; (ii) Pregnant or lactating women; (iii) Refusal to sign informed consent.

The collected information included patients’ clinical data (age, sex, region), department and sample type, mNGS test results, as well as clinical outcomes (**Table 1**). This study was approved by the Ethics Committee of Shanghai Xuhui Central Hospital (Approval Number: 2025041). Informed consent was obtained from all participants for the use of their clinical data and samples.

### 2.2 Sample collection

We analyzed respiratory infection patients who underwent mNGS testing within 5 days after admission. The samples collected had to meet the following minimum volume requirements: at least 4 mL of bronchoalveolar lavage fluid and at least 2 mL of sputum. All specimens were collected aseptically to prevent contamination. After collection, samples were transported to the laboratory for processing as soon as possible. If immediate processing was not possible, samples were stored at -20 °C to prevent DNA degradation and ensure result validity.

### 2.3 Metagenomic next-generation sequencing

According to the kit instructions, DNA was extracted from bronchoalveolar lavage fluid and sputum samples using the TIANamp Micro DNA Kit (DP710-T2A, TIANGEN BIOTECH). The concentration and purity of the DNA were measured using a Qubit 3.0 fluorometer (Thermo Fisher Scientific, Waltham, MA, USA). To construct the sequencing libraries via a shotgun metagenomic strategy, the extracted DNA was sheared into 200–300 bp fragments, followed by end repair. Illumina-compatible sequencing adapters were ligated to the DNA fragments, and PCR amplification was performed to enrich the libraries. Library quality and fragment size distribution were assessed using an Agilent 2100 Bioanalyzer (Agilent Technologies, CA, USA) to confirm the expected insert-size distribution and the absence of adapter dimers. The qualified libraries were sequenced in single-end 75 bp mode on the Illumina NextSeq 550 platform (Illumina Inc., USA).

Raw sequencing data were processed using Trimmomatic v0.36. Quality control criteria included: (i) removal of adapters; (ii) trimming of low-quality bases (quality score < Q20); (iii) removal of reads with N bases > 5%; and (iv) filtration of reads shorter than 50 bp (Bolger et al., 2014). PCR duplicates were removed to minimize amplification bias (Bolger et al., 2014). The resulting clean reads were aligned to the human reference genome GRCh37 using Bowtie v2.2.6 to filter out human genome sequences (Langmead and Salzberg, 2012). To perform taxonomic classification, a genome database built from approximately 27,000 species and 51,543 genomes from NCBI (https://ftp.ncbi.nlm.nih.gov/genomes/) was used as reference (Kitts et al., 2016). Based on the annotated reads, the taxonomic abundances of phages and bacteria were normalized, and the relative abundance of each taxon in the corresponding sample was calculated.

### 2.4 Taxonomic analysis of the phage community

While initial classification was performed at the species level, results were aggregated to higher taxonomic levels (family for phages, genus for bacteria) to ensure robustness (Ye et al., 2019). For the phage dataset, an explicit taxonomic lineage validation was performed using NCBI Taxonomy. Specifically, all detected taxa were checked for their taxonomic lineage, and only annotations belonging to the viral lineage (Viruses) were retained for subsequent phage screening and abundance profiling; annotations assigned to non-viral lineages (e.g., bacterial or fungal taxa) were excluded. For each sample, read counts were obtained by summing reads assigned to the same phage family or bacterial genus. Viral reads that could not be resolved to the family rank and bacterial reads that could not be resolved to the genus rank were grouped as “Unclassified.” Relative abundance was then calculated to account for sequencing depth variation. For visualization, the top 15 classified phage families were selected based on mean relative abundance, with remaining families merged into the “Others” category.

### 2.5 Statistical analysis

R language (version 4.5.1) was used for processing the raw sequencing data in this study. Age was presented as mean ± standard deviation (SD) and by groups, while other categorical variables were described as proportions. The vegan package was used to calculate the Shannon diversity index, Simpson diversity index, and species richness for each sample; Bray-Curtis distances were calculated based on Hellinger-transformed abundance data and then subjected to principal coordinates analysis (PCoA); the PERMANOVA test (adonis2 function) was employed to assess the explanatory power (R²) and significance (p-value) of grouping factors on community structure. For comparisons of phage abundance, diversity indices, and outcome measures between groups, Mann-Whitney U test and Kruskal-Wallis test were used, and the results from multiple tests were adjusted using FDR correction.

### 2.6 Bioinformatics analysis

Since this study employed single-end sequencing and the quality of metagenomic assembly was limited, taxonomic analysis was mainly performed using raw reads, while functional annotation (such as antibiotic resistance genes and lifestyle prediction) was based on assembled contigs. Host-depleted specific reads were assembled using SPAdes v4.2.0; viral sequence prediction and screening were conducted with VirSorter2 v2.2.4 and PhaMer (PhaBOX v2.1.12) to identify potential virus-related clusters. All phage sequences were de-replicated using CD-HIT (v4.8.1), and representative sequences (pOTUs) from clustering were used for subsequent analysis and annotation (Fu et al., 2012). Quality assessment of assembled results was performed using CheckV v1.0.1. Lifestyle prediction for high-quality phage genomes was performed by PhaTYP (PhaBOX v2.1.12) (Shang et al., 2023). Protein-coding genes within high-quality phage genomes were predicted using Prodigal (Hyatt et al., 2010). The resulting protein sequences were searched against the Comprehensive Antibiotic Resistance Database (CARD) using BLASTP (E-value ≤ 1e-5) to identify putative antibiotic resistance genes (ARGs). For each hit, percentage identity (pident) and query coverage (qcovs) were recorded. To facilitate interpretation, BLASTP matches were classified into four categories: (i) stringent matches (pident ≥ 70%, qcovs ≥ 60%); (ii) typical matches (pident ≥ 50%, qcovs ≥ 40%); (iii) permissive matches (pident ≥ 30%, qcovs ≥ 20%); and (iv) weak similarity (all remaining hits) (Enault et al., 2017). Stringent and typical matches were considered high-confidence ARG candidates, whereas permissive and weak matches were interpreted as distant homologs or partial domain-level similarities (Rajmichael et al., 2025).

## 3 Results

### 3.1 Demographic characteristics

A total of 6,404 subjects were enrolled in this study, including 4,837 bronchoalveolar lavage fluid samples and 1,567 sputum samples. The higher proportion of BALF samples compared to sputum reflects the clinical preference for using BALF to confirm lower respiratory tract infections, especially in severe cases, as it helps minimize contamination. The baseline demographic characteristics are shown in **Table 1**.

**Table 1:** Demographic characteristics of BALF and sputum samples.

Variable	BALF group (%)	Sputum group (%)	P value
**Mean Age (Standard Deviation), years**	58.14(14.48)	62.21(13.59)	
**Age Group, years**			<0.001
18-30	257 (5.3)	46 (2.9)	
31-50	922 (19.1)	216 (13.8)	
>50	3658 (75.6)	1305 (83.3)	
**Gender**			<0.001
Male	3102 (64.1)	1135 (72.4)	
Female	1735 (35.9)	432 (27.6)	
**Region**			
East China	1831 (37.9)	928 (59.2)	<0.001
South China	291 (6)	93 (5.9)	
West China	985 (20.4)	169 (10.8)	
North China	1517 (31.4)	347 (22.1)	
Central China	213 (4.4)	30 (1.9)	
**Department**			<0.001
EICU	176(3.6%)	75(4.8%)	
ICU	589(12.2%)	553(35.3%)	
KICU	3(0.1%)	0(0.0%)	
MICU	26(0.5%)	3(0.2%)	
NSICU	17(0.4%)	14(0.9%)	
RICU	666(13.8%)	70(4.5%)	
SICU	118(2.4%)	15(1.0%)	
Rheumatology and Immunology	16(0.3%)	3(0.2%)	
Liver ICU	5(0.1%)	7(0.4%)	
Infectious Diseases	227(4.7%)	29(1.9%)	
Orthopedics	1(0.0%)	1(0.1%)	
Respiratory Medicine	2578(53.3%)	309(19.7%)	
Emergency Department	141(2.9%)	59(3.8%)	
Endocrinology	6(0.1%)	1(0.1%)	
Internal Medicine	43(0.9%)	8(0.5%)	
Neurosurgery	11(0.2%)	77(4.9%)	
Neurology ICU	4(0.1%)	5(0.3%)	
Neurology	25(0.5%)	42(2.7%)	
Nephrology	12(0.2%)	3(0.2%)	
Urology	5(0.1%)	2(0.1%)	
Surgery	9(0.2%)	44(2.8%)	
Gastroenterology	2(0.0%)	2(0.1%)	
Cardiology	2(0.0%)	0(0.0%)	
Cardiac Surgery ICU	16(0.3%)	105(6.7%)	
Cardiac Surgery	6(0.1%)	33(2.1%)	
Thoracic Surgery ICU	3(0.1%)	0(0.0%)	
Thoracic Surgery	26(0.5%)	4(0.3%)	
Hematology	68(1.4%)	81(5.2%)	
Oncology	36(0.7%)	22(1.4%)	
**Total**	4837	1567	

The age distribution of the sample sources ranged widely (18–80 years). The average age of patients in the sputum group was higher (62.21 ± 13.59 years), while the BALF group was younger (58.14 ± 14.48 years). There was a statistically significant difference in age distribution between the two groups (p < 0.05). Additionally, the sex composition also differed: males accounted for 64.1% (n = 3,102) in the BALF group and 72.4% (n = 1,135) in the sputum group, with a statistically significant difference between groups (p < 0.05). Given the retrospective design of this study, this notable male predominance reflects the real-world epidemiological distribution of patients with confirmed bacterial respiratory infections (Li et al., 2021; Wang et al., 2024). This skew is consistent with previous reports that male sex is a significant risk factor for severe respiratory infections and may be attributable to the regional sex ratio, as well as the higher prevalence of comorbidities and lifestyle factors (e.g., smoking) among males (Klein and Flanagan, 2016; Baskaran et al., 2019).

Statistical analysis also revealed a significant difference in the regional distribution of sample sources between the BALF and sputum groups (p < 0.05). Overall, the majority of subjects came from East China (43.1%, n = 2,759), followed by North China (29.1%, n = 1,864), West China (18.0%, n = 1,154), South China (6.0%, n = 384), and Central China with the lowest proportion (3.8%, n = 243).

A further analysis of the departmental sources of the two sample types showed a clear tendency toward specialization. BALF samples mainly came from the Department of Respiratory Medicine (53.3%, n = 2,578), RICU (13.8%, n = 666), and ICU (12.2%, n = 589). Similarly, sputum samples were mainly from the ICU (35.3%, n = 553) and Department of Respiratory Medicine (19.7%, n = 309), with Cardiac Surgery ICU (6.7%, n = 105) as the third largest source. The above distribution characteristics are highly consistent with the clinical indications and standard collection departments for these sample types.

### 3.2 Differences in phage community composition and diversity across sample types

**Figure 1:**
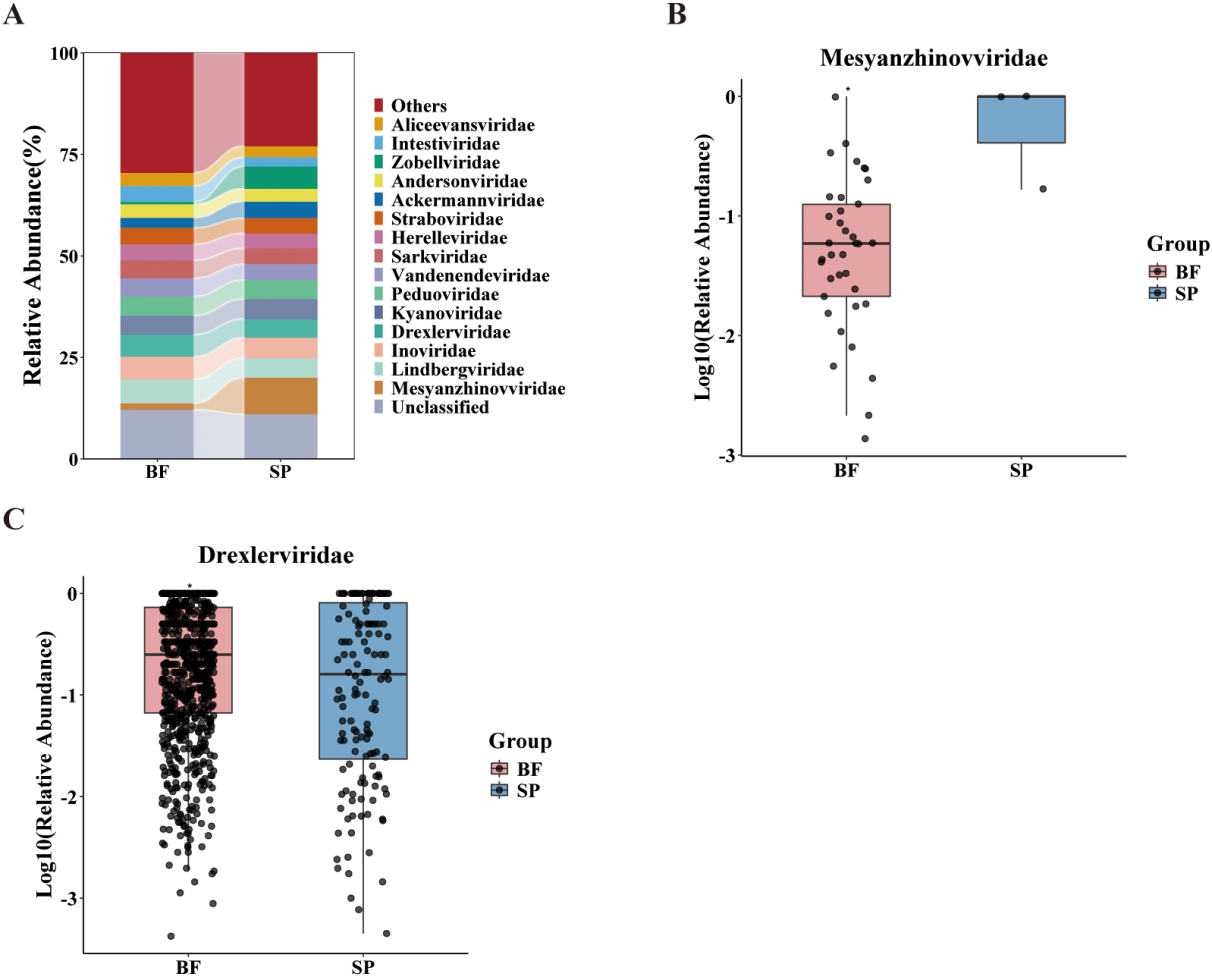
Phage community composition and differential analysis in bronchoalveolar lavage fluid and sputum samples. **(A)** Stacked bar plot showing the relative abundance of the top 15 phage families in BALF and sputum samples. Unclassified sequences are presented as a distinct category, while all remaining low-abundance families are aggregated into “Others.” **(B, C)** Boxplots illustrating the distribution of relative abundances of representative phage families that differed between the two groups of samples.

To investigate the differences in phage community composition and diversity between the BALF group and the sputum group, this study selected the top 15 classified phage families with the highest relative abundance for visualization. Unclassified sequences were presented as a distinct category, while all remaining low-abundance families were aggregated into “Others” (**Figure 1A**). The results revealed distinct inter-group differences in phage community structures; approximately 88% to 89% of phage abundance could be assigned to known families. In the BALF group, the sum of “Others” (29.5%) and unclassified phages (12.1%) accounted for a significant proportion. Among the top 15 classified families, the relative abundance were: *Lindbergviridae* (5.91%), *Inoviridae* (5.56%), *Drexlerviridae* (5.44%), *Kyanoviridae* (4.71%), *Peduoviridae* (4.69%), *Vandenendeviridae* (4.47%), *Sarkviridae* (4.41%), *Herelleviridae* (4.01%), *Straboviridae* (4.00%), *Intestiviridae* (3.92%), *Andersonviridae* (3.32%), *Aliceevansviridae* (3.27%), *Ackermannviridae* (2.49%), *Mesyanzhinovviridae* (1.58%) and *Zobellviridae* (0.57%). Mann-Whitney U testing performed on the top 15 most abundant families (**Figures 1B, C**) indicated that *Mesyanzhinovviridae* (P = 0.02) and *Drexlerviridae* (P = 0.04) showed nominal significance between the groups. Nevertheless, neither retained statistical significance following FDR correction.

**Figure 2:**
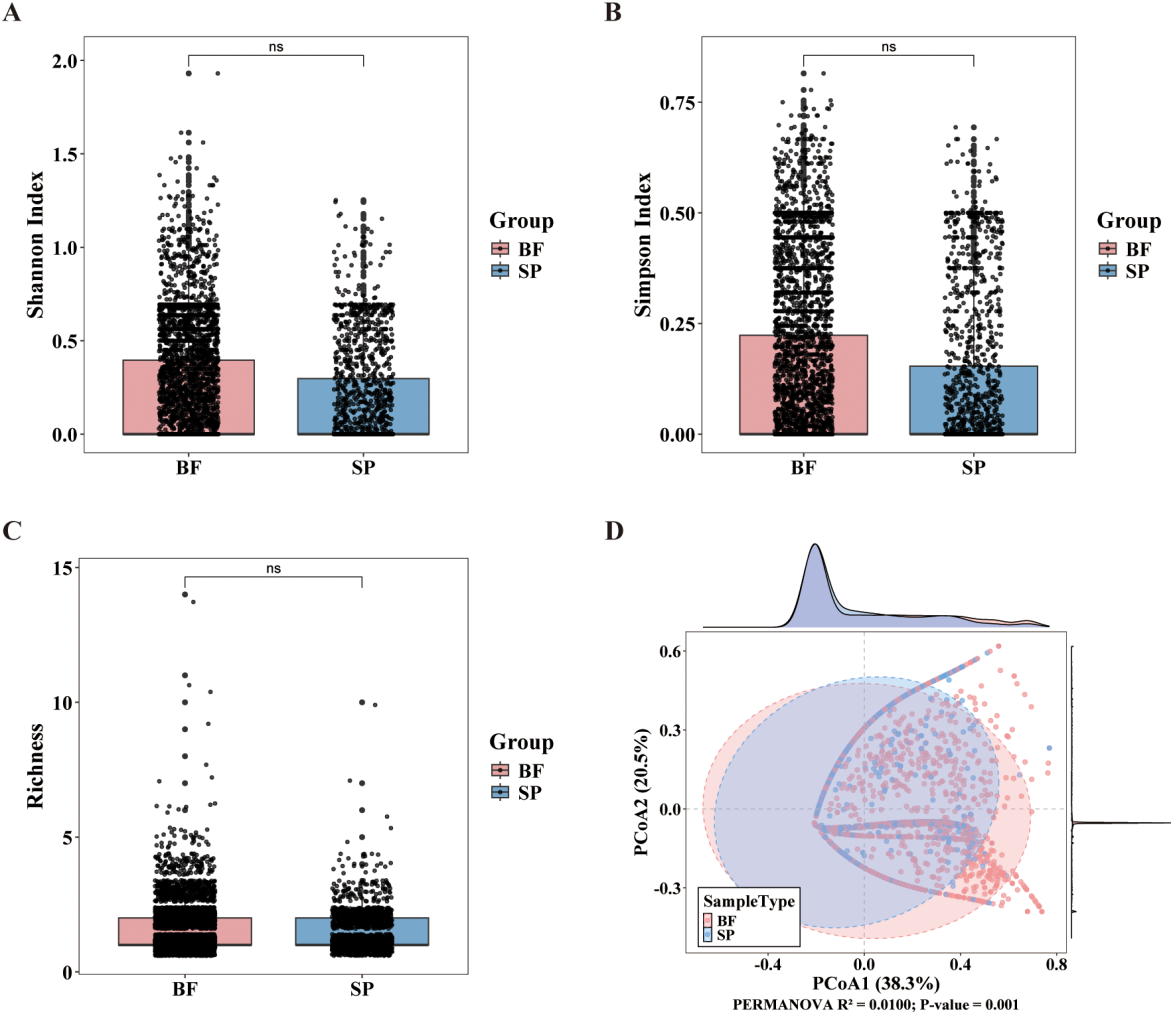
Analysis of phage community diversity between BALF and sputum samples. **(A-C)** Boxplots display phage community diversity indices for the two sample groups, including the Shannon index **(A)**, Simpson index **(B)**, and species richness **(C)**. **(D)** Principal Coordinate Analysis (PCoA) based on Bray-Curtis distance.

Alpha diversity and Beta diversity were assessed for both the BALF and sputum groups (**Figures 2A–D**). The results of the alpha diversity analysis showed no statistically significant differences between the two groups in Shannon diversity index, Simpson evenness index, and richness (p > 0.05), indicating comparable diversity and evenness within the microbiomes of both groups. Principal Coordinates Analysis (PCoA) based on Bray-Curtis distances indicated a significant difference in phage community structures between the two sample groups (R² = 0.0100, P = 0.001). In summary, although phage diversity and evenness were similar in the two groups, the composition and distribution of species differed significantly.

### 3.3 Analysis of phage community diversity in two sample types between ICU and non-ICU patients

Based on sputum and bronchoalveolar lavage fluid samples, patients were divided into ICU and non-ICU groups according to their ward source. The sample sizes were as follows: BALF (ICU group/non-ICU group: 1623/3214) and sputum (846/721).

**Figure 3:**
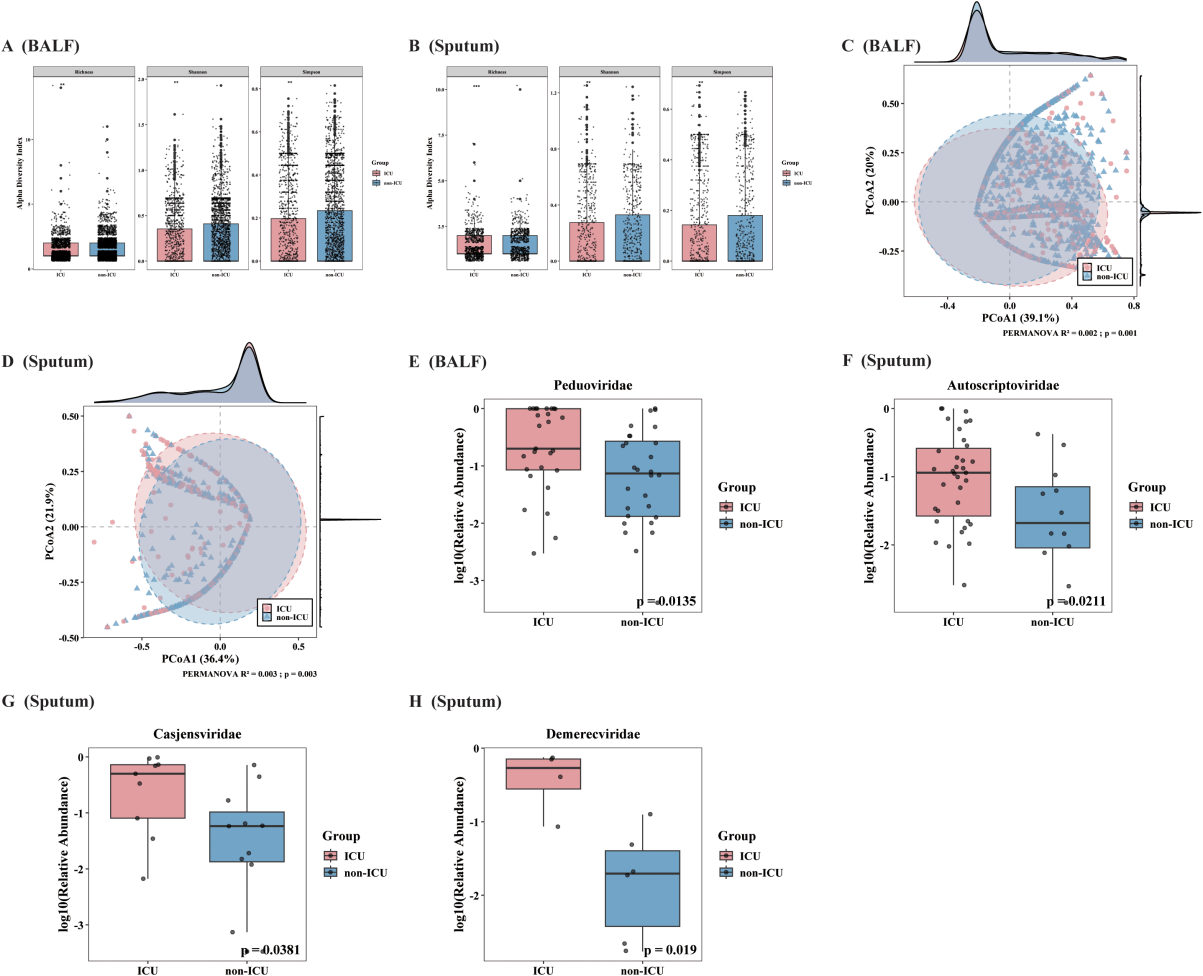
Comparison of phage community diversity and abundance distribution between ICU and non-ICU patients across two clinical sample types. **(A, B)** Comparison of phage alpha diversity indices (Shannon, Simpson, Richness) between ICU and non-ICU groups in two clinical sample types [**(A)** BALF, **(B)** sputum]. **(C, D)** Principal Coordinates Analysis (PCoA) based on Bray-Curtis distances shows the community structure distribution of phages in ICU and non-ICU groups for both sample types [**(C)**: BALF, **(D)** sputum]. **(E-H)** Relative abundance distributions of phage families with significant differences between ICU and non-ICU groups identified by Wilcoxon rank-sum test. **(E)** show *Peduoviridae* in BALF samples; **(F–H)** show *Autoscriptoviridae*, *Casjensviridae*, and *Demerecviridae* in sputum samples.

Alpha and Beta diversity analyses were used to evaluate differences in the phage community structure between ICU and non-ICU patients across different clinical sample types. The results showed consistent and significant differences in the phage communities between the ICU and non-ICU groups in both types of respiratory samples. Specifically, the Alpha diversity in the non-ICU group was significantly higher than that in the ICU group (**Figures 3A, B**). Furthermore, Beta diversity analysis confirmed (**Figures 3C, D**) that the overall community structures between the two groups were statistically significantly separated (BALF: R² = 0.002, P = 0.001; sputum: R² = 0.003, P = 0.003).

Further analysis was conducted to explore the differences in phage community distribution between the ICU and non-ICU groups by comparing the relative abundance of phage families in both sample types using the Mann-Whitney U test (**Figures 3E–H**). In the BALF group, significant differences in the relative abundance of phage families were observed between ICU and non-ICU patients, with *Peduoviridae* notably showing a higher average abundance in the ICU group (p=0.013). In the sputum group, three phage families exhibited significant differences in abundance between the ICU and non-ICU groups. The relative abundance of the *Autoscriptoviridae* family was significantly higher in the ICU group (p = 0.021); the *Casjensviridae* (p = 0.038) and *Demerecviridae* (p = 0.019) families were also significantly more abundant in the ICU group compared to the non-ICU group.

The above results suggest that in both sputum and BALF samples, the abundance distribution of certain phage families is associated with patients’ ICU admission status. Notably, the families *Autoscriptoviridae*, *Casjensviridae*, *Demerecviridae*, and *Peduoviridae* all exhibit higher abundances in the ICU group. In summary, this study found a marked decrease in phage community diversity and significant structural changes in the respiratory tract of ICU patients, with some phage families displaying increased abundance in ICU samples. These findings indicate a close relationship between ICU admission status and the composition and distribution of respiratory tract phage communities.

### 3.4 Association between phage communities and patient clinical outcomes

To investigate the potential association between phage community characteristics and patient outcomes, this study integrated phage metagenomic data from 73 BALF samples and 41 sputum samples. Combined with clinical information on patient outcomes—including cure, improvement, non-recovery, and death—we systematically analyzed differences in phage community structures among groups with different treatment outcomes.

**Figure 4:**
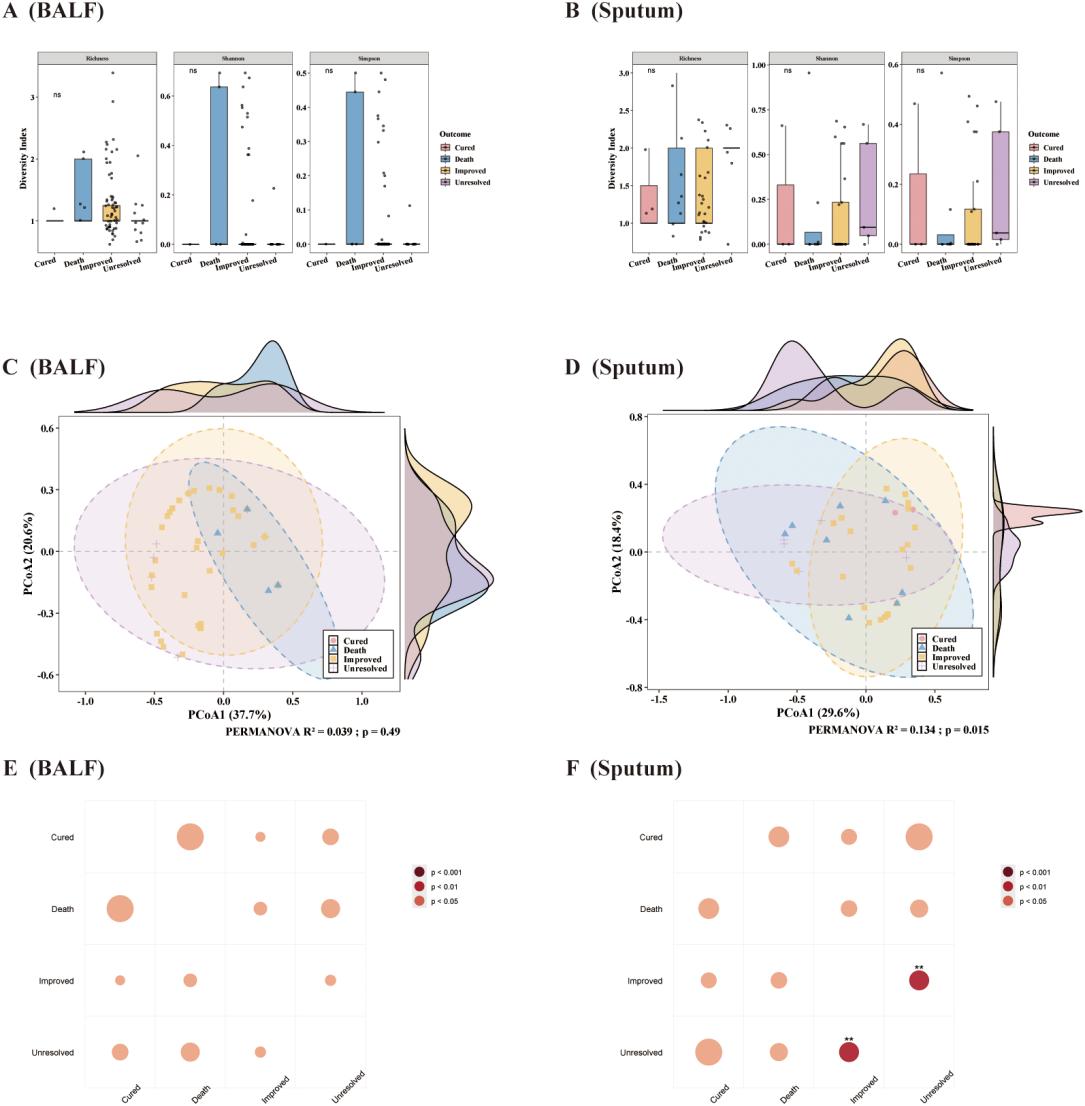
Comparison of phage community diversity and structure among prognosis groups for the two sample types. **(A, B)** Comparison of phage alpha diversity indices (Shannon, Simpson, Richness) among different prognosis groups [**(A)** BALF, **(B)** sputum]. **(C, D)** Principal coordinate analysis (PCoA) based on Bray-Curtis distance and PERMANOVA results, showing differences in phage community structure among prognosis groups [**(C)** BALF, **(D)** sputum]. **(E, F)** Pairwise PERMANOVA bubble plots comparing prognosis groups in the two sample types [**(E)** BALF, **(F)** sputum]; bubble size represents R² value explaining the variance, and color as well as asterisks indicate statistical significance (p < 0.05, p < 0.01, p < 0.001).

Alpha diversity analysis was conducted among different prognosis groups (**Figures 4A, B**). The results showed that, regardless of whether the samples were bronchoalveolar lavage fluid or sputum, there were no statistically significant differences in the alpha diversity indices (Richness, Shannon, Simpson) between prognosis groups. Principal coordinate analysis (PCoA) based on Bray-Curtis distance indicated a noticeable tendency for community structure separation among the four prognosis groups (**Figures 4C, D**). PERMANOVA analysis revealed that the phage community structure in bronchoalveolar lavage fluid samples was not significantly associated with clinical prognosis (R² = 0.039, p = 0.481); in contrast, the phage community structure in sputum samples was significantly associated with clinical prognosis (R² = 0.134, p = 0.014). Further pairwise PERMANOVA analysis among the prognosis groups of sputum samples indicated statistically significant differences in phage community structure only between the Improved and Unresolve groups (**Figure 4F**). Finally, for both types of samples, the Kruskal-Wallis test was applied to evaluate differences in the abundance of each phage family among prognosis groups. The results showed that the abundance of the vast majority of phage families did not differ significantly across prognosis groups (all adjusted p-values were greater than 0.05). This suggests that changes in phage community abundance may not be the main factor affecting prognosis, or that the sample size may be insufficient to detect subtle differences.

### 3.5 Relationship between phages and bacteria

To investigate group-specific correlations between bacteria and phages, Spearman correlation analyses were performed separately for BALF and sputum samples to assess the associations between the abundance of bacterial genera and phage families.

**Figure 5:**
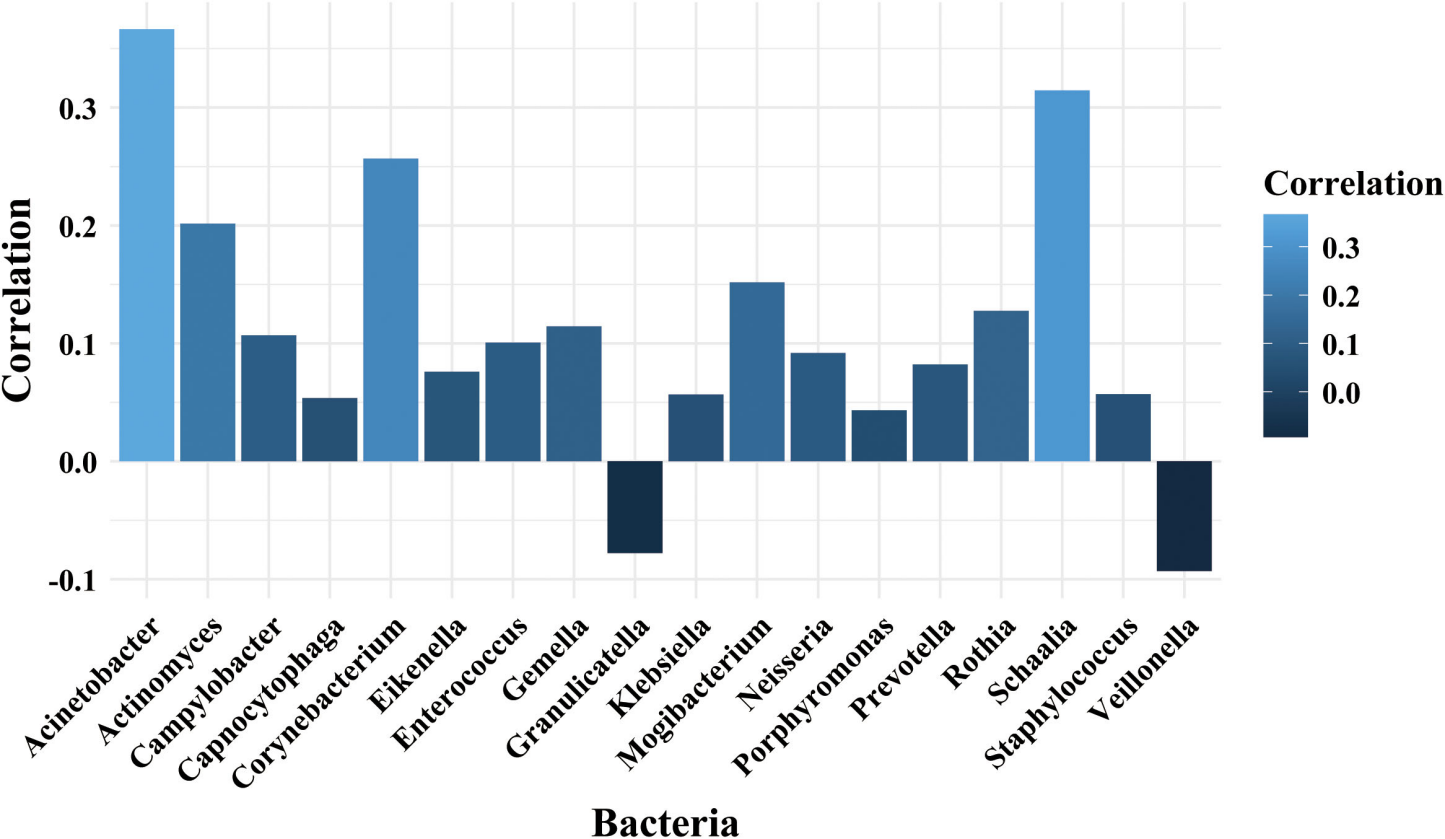
Spearman correlation analysis between the *Aliceevansviridae* phage family and bacterial genera in sputum samples. The bar chart displays the correlation coefficients for bacterial genera that showed statistically significant associations (p < 0.05) with *Aliceevansviridae* in the sputum group. The color gradient represents the strength of the correlation. Note: This specific interaction network involving *Aliceevansviridae* was observed exclusively in the sputum samples; no significant clustering of any specific phage family was found in the BALF group.

In the sputum samples, the analysis identified 35 significant correlations (p < 0.05). Positive correlations dominated the network. Notably, the *Aliceevansviridae* family exhibited extensive associations with multiple bacterial genera. Among them, a total of 18 bacterial genera were found to be correlated with the *Aliceevansviridae*. The strongest correlations were observed between *Aliceevansviridae* and *Acinetobacter* (r = 0.366), *Schaalia* (r = 0.315), and *Corynebacterium* (r = 0.257), as visualized in **Figure 5**. Additionally, unclassified phages showed significant positive correlations with genera such as *Klebsiella* and *Granulicatella*.

In the BALF samples, the interaction network was distinct and notably weaker. We identified 21 significant correlations, nearly all of which involved unclassified phages. These unclassified phages showed weak but significant positive correlations with dominant pathogens, including *Klebsiella* (r = 0.123), *Streptococcus* (r = 0.106), and *Rothia* (r = 0.098). Unlike in sputum, no specific classified phage families formed significant interaction clusters with bacteria in the BALF group.

### 3.6 Analysis after sequence assembly

The abundance of phage families and bacterial genera in the above study showed certain correlations, suggesting complex ecological interactions may exist between phages and bacteria in the microbiota of clinical samples. However, due to the limitations of read length in single-end sequencing data and the complex microbial background, read-level analyses have certain constraints regarding taxonomic resolution and functional annotation, making it difficult to deeply explore the potential functions of phages and their interaction mechanisms with bacteria. To systematically examine the ecological links between phages and bacteria—especially their lifestyles in respiratory tract infection samples—and to assess the potential role of phages as vectors for antibiotic resistance genes in the spread of bacterial resistance, this study further selected 194 samples with relatively high sequencing depth and data quality for metagenomic *de novo* assembly. This subset includes 91 BALF samples and 103 sputum samples. The goal is to leverage contig-level analysis to supplement the limitations of the read-level approach and more precisely reveal the functional features of phages and their ecological roles.

First, quality control was conducted on the raw FASTQ files using fastp software (v0.23.4), and Bowtie2 was used to identify and remove human host-derived sequences. Subsequently, SPAdes was employed for *de novo* metagenomic assembly of the non-host reads, while phage identification was comprehensively performed using VirSorter2 and PHAMER. CD-HIT was utilized to de-replicate and cluster phage sequences, resulting in a total of 1,768 pOTUs, ranging in length from 3,001 bp to 136,723 bp, with an average length of 9,389.32 bp. To assess the completeness and quality of the phage genomes, all viral sequences were analyzed using CheckV (v1.0.1). Among the 1,768 viral sequences obtained, 18 (1.0%) were complete, 38 (2.1%) were high-quality, 126 (7.1%) were medium-quality, 1,394 (78.8%) were low-quality, and 192 (10.9%) could not be determined. Subsequent analyses were all conducted based on medium-quality, high-quality, and complete sequences (a total of 182 sequences, accounting for 10.3% of all sequences).

**Figure 6:**
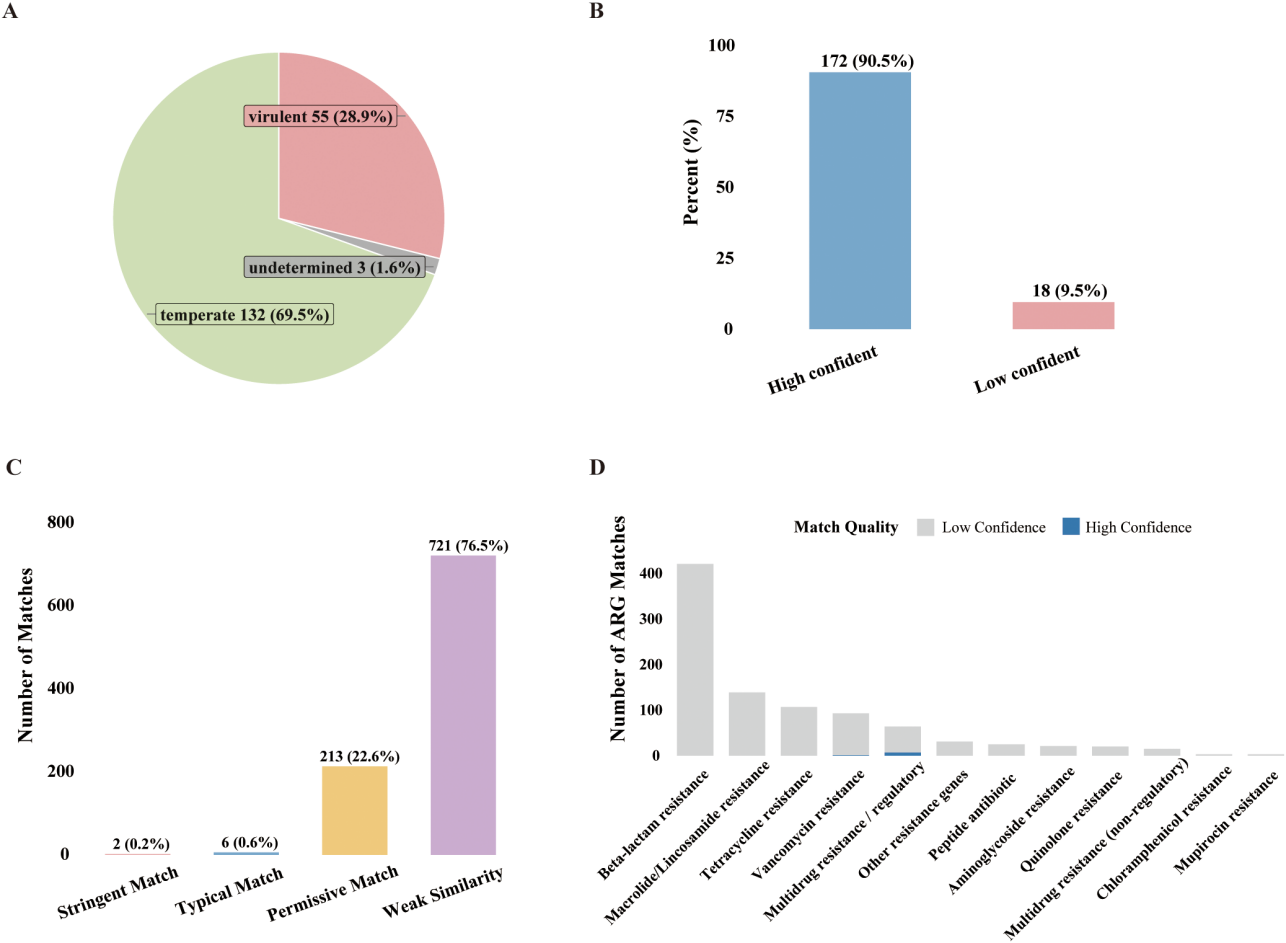
Phage lifestyle prediction and antimicrobial resistance gene risk assessment. **(A)** Lifestyle classification results. **(B)** Distribution of prediction confidence scores for lifestyle. **(C)** Grading of match quality for phage antimicrobial resistance genes. **(D)** Abundance and quality stratification of resistance categories. The stacked bar chart distinguishes between high-confidence matches (Stringent and Typical matches, blue) and low-confidence homologs (Permissive and Weak matches, grey).

The life cycle types of 190 phages were predicted using the PhaTYP model (PhaBOX v2.1.12). The results showed that 98.4% of the sequences could be successfully classified, of which 69.5% (n=132) were predicted to be temperate and 28.9% (n=55) were predicted to be virulent (**Figure 6A**). Prediction confidence was evaluated based on PhaTYP scores, with a score ≥0.9 considered high confidence. The results indicated that 90.5% (n=172) were high-confidence predictions, demonstrating the reliability of the model; the remaining 9.5% (n=18) were low-confidence predictions (**Figure 6B**). Additionally, temperate phages showed a higher proportion of high-confidence predictions (93.9% vs 87.3%), potentially reflecting a greater taxonomic distinctiveness in their sequence characteristics.

In this study, we analyzed protein sequences computationally predicted from 59 high-quality phage genomes against the CARD database using the official ARO index, identifying a total of 942 putative antibiotic resistance gene (ARG) matches. As shown in **Figures 6C**, the vast majority of matches (99.2%) were classified as distant homologs with low confidence. Specifically, only 2 matches (0.2%) met the strict threshold, and another 6 matches (0.6%) were classified as Typical Matches. The remaining matches fell into the Permissive (22.6%) and Weak Similarity (76.5%) categories, indicating that the detected hits were predominantly low-identity homologs lacking definitive functional evidence. Regarding the distribution of resistance categories (**Figure 6D**), Beta-lactam resistance was the most abundant category, accounting for 44.7% (421/942) of the total matches, followed by Macrolide/Lincosamide resistance (14.8%) and Tetracycline resistance (11.4%). However, stratification by match quality revealed that these high-abundance Beta-lactam hits consisted almost exclusively of low-confidence homologs. Overall, no conclusive evidence of high-risk ARGs belonging to key clinical families in the CARD database was found (**Supplementary Figure 1**).

## 4 Discussion

This study systematically revealed the composition and clinically relevant characteristics of phage communities in BALF and sputum samples from patients with respiratory infections, based on metagenomic next-generation sequencing technology. The results showed no statistically significant differences in alpha diversity metrics between the two types of clinical samples (p > 0.05); however, their community structures differed significantly. Further analysis indicated that the characteristics of phage communities in the respiratory tract were not only significantly associated with whether patients were admitted to the ICU, but also closely linked to clinical outcomes. This suggests that phage communities could serve as potential microbial biomarkers.

Alpha diversity analysis revealed no significant differences between the groups in the Shannon diversity index, Simpson evenness index, or species richness (p > 0.05), indicating that, in this study, the sample type had a generally consistent impact on the diversity, evenness, and richness of respiratory phage communities. However, principal coordinate analysis (PCoA) based on Bray–Curtis distances demonstrated significant differences in phage community structures between the two groups. These differences may reflect the admixture of oral and nasal microbiota in sputum samples, as well as the distinct microenvironments and host bacterial distributions in the upper versus lower respiratory tracts (Bassis et al., 2015; Huffnagle et al., 2017; Man et al., 2017). In addition, given the retrospective design of this study, strict clinical matching between the BALF and sputum groups was not feasible. Therefore, the observed variation in community structure likely represents the combined effects of anatomical distinctions and the inherent baseline heterogeneity in patient characteristics within these real-world cohorts. Despite differences in community structure, the core phage composition of the two sample groups exhibited a high degree of consistency. Overall, the phage communities in both BALF and sputum were dominated by a similar set of bacteriophage families, including *Inoviridae*, *Mesyanzhinovviridae*, *Zobellviridae*, *Drexlerviridae*, *Peduoviridae*, and *Ackermannviridae*. Moreover, a high proportion of unknown and unclassified phages was identified in both sample types, indicating the presence of a rich diversity of previously unrecognized or unidentified phage species in respiratory samples (Conradie et al., 2024; Purcell et al., 2025). Among them, the high abundance of the *Inoviridae* family detected in BALF samples and the *Mesyanzhinovviridae* family found in sputum samples are consistent with previous findings regarding phages in respiratory infections (Foxall et al., 2024; Roux et al., 2019). Previous studies have shown that members of the family *Inoviridae* typically utilize Gram-negative bacteria, such as *Pseudomonas aeruginosa*, as hosts and are involved in biofilm formation during chronic lung infections (Foxall et al., 2024; Knezevic and Adriaenssens, 2021). Similarly, families such as *Drexlerviridae*, *Peduoviridae*, and *Ackermannviridae* are widely known to infect Enterobacteriaceae and other Gram-negative bacilli, including *Escherichia*, *Klebsiella*, and *Salmonella* (Cranston et al., 2022; Casjens and Grose, 2016; Sørensen and Brøndsted, 2024), whereas *Herelleviridae* predominantly target Gram-positive bacteria such as *Bacillus* (Kornienko et al., 2023). For several recently defined families (e.g., *Mesyanzhinovviridae*, *Zobellviridae*, and *Aliceevansviridae*), although experimental characterization remains limited, genomic evidence and host prediction analyses suggest that they also tend to infect opportunistic pathogens such as *Pseudomonas* and *Streptococcus* (Pchelin et al., 2024; Troshin et al., 2025). Although a systematic quantitative analysis of bacterial abundance was not performed in this study, existing literature indicates that pathogens such as *Pseudomonas*, *Klebsiella*, and *Acinetobacter* are frequently present in samples from respiratory tract infections (Bart et al., 2021; Reyes et al., 2025). The high clinical prevalence of these pathogens and their status as established hosts provide a plausible biological basis for the observed high abundance of the associated phage families in both sample types.

In both types of samples, there were significant differences in phage diversity and community structure between the ICU and non-ICU groups. Specifically, phage diversity was markedly reduced in ICU patients, and the community structure differed significantly from that of the non-ICU group. This phenomenon may be closely related to changes in the physiological state, treatment strategies, and microbial ecological environment in critically ill patients (Chang et al., 2023; Hyblova et al., 2023). On one hand, ICU patients often experience immune imbalance, enhanced inflammatory responses, and disruptions to the host microbiota, all of which can affect the phage–bacteria host relationship, ultimately leading to reduced phage diversity (Singer et al., 2016; Cheng et al., 2024). On the other hand, clinical interventions such as the use of broad-spectrum antibiotics may also alter the bacterial community structure and indirectly impact the phage community through disturbances in the host microbiome (Wahida et al., 2021; Zhou et al., 2022; Wang et al., 2023). Our findings further reveal that, in addition to an overall decline in diversity, specific phage families (*Peduoviridae*, *Autoscriptoviridae*, *Casjensviridae*, and *Demerecviridae*) were significantly more abundant in ICU patients. Most of these families belong to the tailed bacteriophages (class *Caudoviricetes*) and have been reported to infect common clinical pathogens, including *Enterobacteriaceae* and *Pseudomonadaceae* (Casjens and Grose, 2016; Chakraborty et al., 2024). The enrichment of these phage families in ICU samples may have the following explanations. First, it may primarily reflect an increased burden of their bacterial hosts during severe infection and ICU-related dysbiosis (Ferravante et al., 2022a). Second, many *Caudoviricetes* are temperate phages capable of lysogeny. These prophages can regulate bacterial gene expression or carry accessory genes, affecting bacterial survival and virulence (Bowring et al., 2022; Davies et al., 2016). In addition, phages can transfer virulence factors and antimicrobial resistance genes between bacteria through transduction (Watson et al., 2021). In ICU settings, antibiotic exposure can induce prophages and increase this gene transfer (Gavric and Knezevic, 2026). These mechanisms suggest that the enriched phage families in the ICU may promote the expansion of bacteria associated with severe infections or be involved in infection development (Chavignon et al., 2022; Ferravante et al., 2022a; Kolupaeva et al., 2025).

The structure of the phage community in sputum samples was significantly associated with patient clinical outcomes, whereas a similar trend was not observed in bronchoalveolar lavage fluid samples. This may be because bronchoalveolar lavage fluid primarily reflects the lower respiratory tract microenvironment, while sputum captures both oropharyngeal and upper respiratory tract information (Peng et al., 2020; Miao et al., 2022). As a result, there are differences in microbial composition and dynamic changes between the two, and the overall microbial load is generally higher in sputum, making it easier to detect phage ecological differences related to the host’s condition (Durack et al., 2020; He et al., 2022). Further pairwise comparisons of sputum samples revealed statistically significant differences in community structure only between the Improved and Unresolved groups. However, there were no phage families with specific changes in abundance among the outcome groups. This suggests that the differences among prognostic groups may be driven by cooperative shifts in multiple low-abundance phages or by more complex host-phage interaction patterns, rather than being caused by a single dominant phage group (Mirzaei and Maurice, 2017; Shkoporov and Hill, 2019). In addition, previous studies have shown that phages can influence bacterial diversity and antibiotic resistance through mechanisms such as lysis and gene transfer, thereby indirectly affecting infection outcomes and treatment efficacy (Clemente et al., 2012; Wahida et al., 2021). Therefore, alterations in phage community structure or enrichment of certain phage groups, particularly within the upper respiratory tract microecology, may be closely related to patient clinical prognosis.

The correlation analysis revealed phage-bacteria interaction patterns between the upper and lower respiratory tracts. In sputum, the robust network centered on *Aliceevansviridae* and genera like *Acinetobacter* and *Schaalia* suggests that these associations may function as an ecologically interactive unit, collectively contributing to shifts in the respiratory microenvironment. The association of *Aliceevansviridae* (a phage family known to infect *Streptococcus*) with common oral bacteria like *Gemella* and *Schaalia*, which share a similar oral environment, biologically validates the phage–bacteria co-occurrence patterns observed in our upper respiratory samples (Pchelin et al., 2024). In contrast, the BALF microbiome exhibited a simplified correlation pattern dominated by unclassified phages and potential pathogens. This observation is likely attributable to the inherently lower microbial biomass of the lower respiratory tract and the dilution of microbes during BALF collection (Dionísio, 2012; Dickson et al., 2016). Furthermore, current viral databases (e.g., RefSeq, IMG/VR) exhibit certain biases, lacking comprehensive data on lung-specific lineages compared to oral or gut phages, which suggests that a significant portion of the lung phageome consists of unclassified or unknown viral sequences (Liang and Bushman, 2021).

Limited by single-end mNGS sequencing data and assembly depth, the taxonomic identification accuracy for viruses after assembly remains insufficient to comprehensively analyze phage community diversity and abundance. Therefore, this study utilized direct read-based annotation for taxonomic classification and abundance statistics, while assembly analysis focused on lifestyle prediction and antibiotic resistance gene (ARG) screening. Lifestyle prediction results showed that temperate phages dominate in body fluid samples, with higher taxonomic confidence. This distribution pattern is consistent with previous reports regarding phage lifestyles under disease conditions (Clooney et al., 2019; Avellaneda-Franco et al., 2023). The predominance of temperate phages may help maintain microbiome stability by facilitating gene exchange and ecological adaptability (Avellaneda-Franco et al., 2023; Tang et al., 2025). Notably, the proportion of temperate phages further increased in disease states, whereas the proportion of virulent phages decreased, indicating that shifts in phage lifestyle may be involved in disease progression and microecological imbalance (Obeng et al., 2016; Silveira and Rohwer, 2016; Hu et al., 2021). ARG screening results revealed that among the 942 CARD matches identified in this study, the vast majority (99.2%) exhibited low similarity to known antibiotic resistance genes. Combined with the distribution characteristics of resistance categories—where high-abundance categories such as Beta-lactam resistance are composed almost exclusively of distant homologs—these data suggest that the phage genomes recovered from respiratory bacterial infection samples in this study may not constitute a major reservoir of known, high-confidence ARGs (Enault et al., 2017). However, findings based solely on sequence similarity to current databases cannot exclude the presence of divergent or uncharacterized resistance determinants. Therefore, we interpret these results as an initial, sequence-based risk assessment; while they indicate a low genetic burden of resistance potential, they do not constitute definitive proof of biological safety in the absence of phenotypic confirmation.

However, this study still has some limitations. First, although the sampled sources covered 21 provinces and cities across China, the geographic distribution remains relatively concentrated, and all data were obtained using mNGS technology, which could introduce certain selection bias. Second, our sequencing approach relied solely on DNA extraction, meaning RNA phages were not evaluated. Therefore, the exclusion of RNA phages (such as *Leviviridae* and *Cystoviridae*) may lead to an underestimation of total viral diversity and incomplete analysis of their bacterial hosts (Bollback and Huelsenbeck, 2001; Alipour-Khezri et al., 2024). This omission further limits the evaluation of phage-mediated bacterial abundance, metabolism, and antibiotic resistance (Callanan et al., 2018; Bae et al., 2023). Third, the clinical data collection was limited to basic demographics, lacking comprehensive laboratory records—specifically antibiograms. This absence prevented a direct correlation between phage-borne ARG candidates and bacterial resistance phenotypes. Consequently, our ARG analysis reflects genomic potential only; future studies integrating matched genomic and susceptibility data are required to assess clinical impact. Finally, as a retrospective observational study, our work can only reveal associations between the phage community and clinical phenotypes, without capturing dynamic changes during disease progression or establishing causality.

Despite the aforementioned limitations, this study presents a two-year retrospective analysis mapping the associations between phage communities and key clinical phenotypes in Chinese patients with respiratory tract infections. The findings suggest that future prospective studies should dynamically collect samples at multiple time points, incorporate data such as antibiotic usage history, host immune indicators, and metatranscriptomics, and integrate phage characteristics with clinical parameters to develop microbial biomarker models for infection diagnosis or prognostic risk assessment.

## 5 Conclusion

In summary, this study demonstrates the crucial role of metagenomic next-generation sequencing in elucidating the complexity of phage communities in bronchoalveolar lavage fluid and sputum. The results reveal the dynamic changes of phage communities in patients with respiratory tract infections and their clinical significance, uncovering a significant association between ICU admission and respiratory tract phage community alterations, thereby enriching the theoretical foundation of infectious microecology. Future research should employ prospective cohort designs and integrate multi-omics and functional experiments to further explore the specific roles and mechanisms of key phages in disease progression, promoting their translation into clinical applications.

## Data Availability

The raw metagenomic data generated in this study have been deposited in the Genome Sequence Archive (GSA) at the National Genomics Data Center (NGDC), China National Center for Bioinformation / Beijing Institute of Genomics, Chinese Academy of Sciences (GSA: CRA039027), and are publicly accessible at https://ngdc.cncb.ac.cn/gsa.
