# Supplementary material for: Phage characterization analysis in respiratory samples from infected patients based on metagenomic next-generation sequencing
Source: Front Cell Infect Microbiol. 2026 Mar 12;16:1779296. doi: 10.3389/fcimb.2026.1779296 (PMC13017799; doi:10.3389/fcimb.2026.1779296)
Supplement: Supplementary file 1 [file Table1.docx]

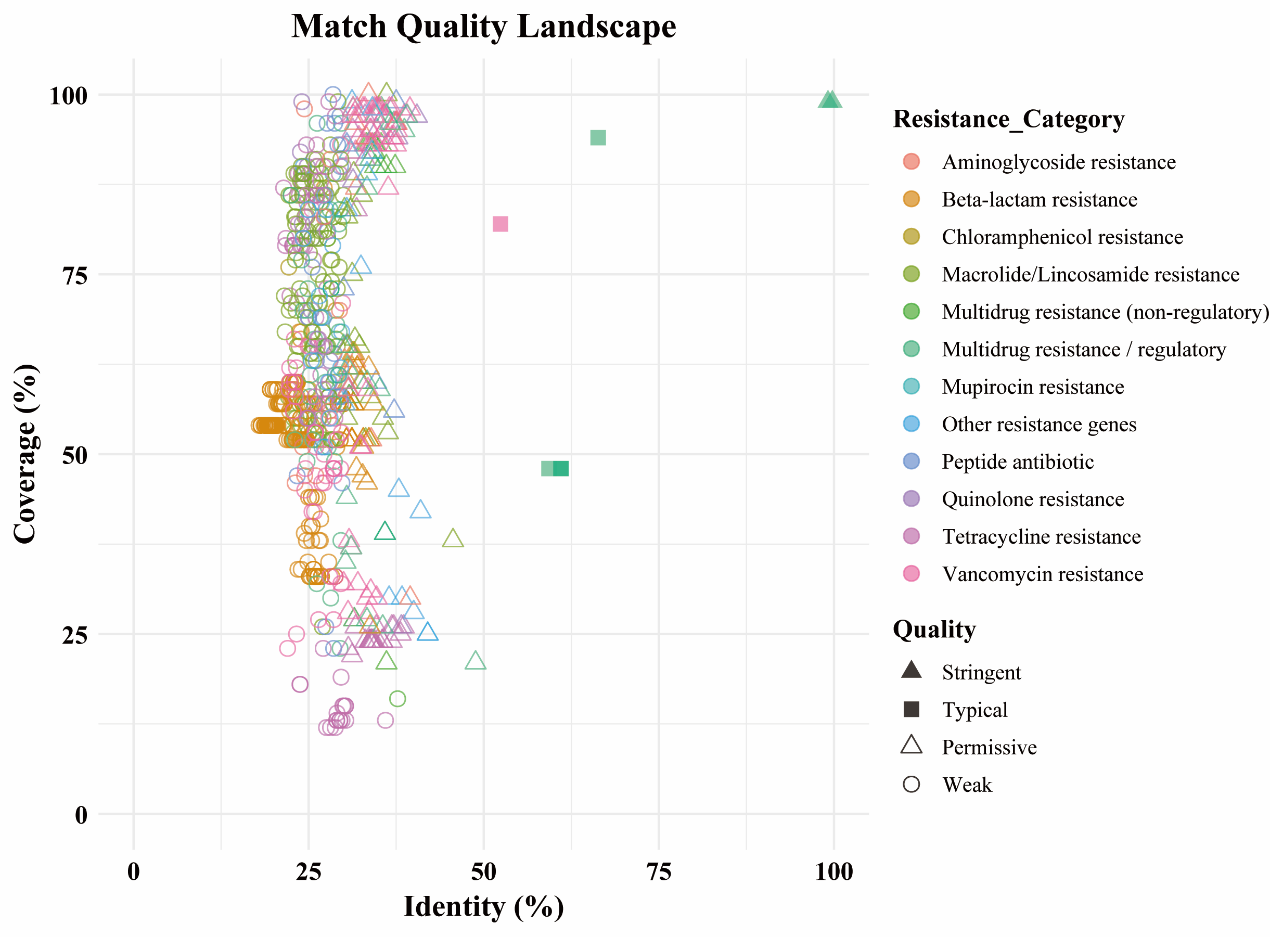


**Figure S1.** Match quality landscape of detected ARG hits.
A scatter plot displaying sequence identity versus query coverage for all 942 detected hits against the CARD database. Points are colored by resistance category and shaped by quality grade (Strict, Typical, Permissive, Weak). The majority of hits cluster in the low-identity/low-coverage region, indicating a lack of high-confidence resistance determinants.
